# Comparative Evaluation of the Inhibitory Effect of Lactobacillus rhamnosus on Halitosis-Causing Bacteria: An Invitro Microbiological Study

**DOI:** 10.7759/cureus.38568

**Published:** 2023-05-05

**Authors:** Aishwarya V Patil, Sarika S Shetty, Ashvini M Padhye

**Affiliations:** 1 Department of Periodontics, Mahatma Gandhi Mission’s Dental College and Hospital, Navi Mumbai, IND

**Keywords:** volatile sulphide compound, tannerella forsythia, prevotella intermedia, porphyromonas gingivalis, halitosis, lactobacillus rhamnosus, probiotics

## Abstract

Aim: To determine the effectiveness of *Lactobacillus rhamnosus* in inhibiting halitosis-causing bacteria relative to other possible inhibitors, such as mouthwashes.

Materials and Methods: This in vitro study was done using a diffusion test with three groups with 11 samples in each group: group A, *Porphyromonas gingivalis*; group B, *Tannerella forsythia*; and group C, *Prevotella intermedia*. At 24, 48, and 72 hours, the inhibitory effect of *L. rhamnosus* was tested.

Results: A statistically significant difference was seen for halo formation in group A, where all 11 samples showed an inhibitory effect after 72 hours. After 48 hours, seven of the 11 samples in group B and nine of the 11 samples in group C showed inhibitory effects.

Conclusion: The study found that *L. rhamnosus* had an inhibitory effect on halitosis-causing bacteria like *P. gingivalis* after 72 hours, which was statistically significant. The same was true for *T. forsythia* and *P. intermedia* after 48 hours. This means that *L. rhamnosus* has an inhibitory effect on halitosis-causing bacteria like *P. gingivalis*.

## Introduction

Microorganisms produce probiotics, which are chemicals that encourage the development of other germs. Lilly and Stillwell, in 1965, termed probiotics as a substance produced by one microorganism that promotes the growth of another microorganism [1]. Fuller studied probiotics and their effects since their discovery in 1989 and defined them as “a live microbial feed supplement that improves the intestinal microbial balance of the host animal” [2]. The World Health Organisation (WHO) established the term “probiotics” in 2006 to describe live microorganisms that promote host health when administered in adequate doses [3]. The most common probiotic bacteria are strains of *Lactobacillus* and *Bifidobacterium*. An improvement in gastrointestinal health and immune modulation is seen with the use of *Lactobacillus rhamnosus*, which is also called *L. rhamnosus* GG. Improvement in clinical parameters was seen in patients with periodontitis after using these probiotics [4-6]. Evidence suggests that if *L. rhamnosus* SP1 is consumed regularly, it has the potential to work for a long time and provide health advantages [7-13]. Probiotics are considered beneficial in periodontal therapy as they serve as anti-inflammatory agents and help reduce periodontal pathogens [7,14,15].

Oral malodour, commonly referred to as “bad breath” and “halitosis,” is a term used to describe an unpleasant smell coming from the mouth [16]. Common causes of oral malodour include gingivitis, areas of plaque stagnation, poor dental hygiene, and tongue coating [17]. In 1977, Tonzetich hypothesised that volatile sulphur compounds (VSCs) like hydrogen sulphide, methyl mercaptan, and dimethyl sulphide were to blame for the foul odour [18]. Oral malodour has been traced back to VSCs generated by bacteria and protein putrefaction of sulphide-containing amino acids. Dry mouth, poor oral hygiene practices, tongue coating, and periodontal disease have all been linked to elevated levels of VSCs [19]. An inflammatory response is induced when VSCs penetrate deep into gingival tissues, alter their permeability, and damage the epithelium, basement membrane, and underlying lamina propria [20,21].

Oral malodour is commonly linked to periodontal pathogens that produce VSCs [19,20]. These include *Porphyromonas gingivalis*, *Tannerella forsythia*, *Prevotella intermedia*, *Fusobacterium nucleatum*, and *Treponema denticola*. Chemical or antimicrobial therapy treats oral malodour by decreasing the bacterial role in the condition, but its long-term use is not without risk. The return of halitosis-causing bacteria after the treatment is discontinued accounts for the short-lived improvement in malodour. Probiotics inhibit the proliferation of bacteria that cause bad breath, so they may be used as a complementary therapy for treating and avoiding bad breath [22]. *Lactobacillus* and *Streptococcus* are considered beneficial bacteria present in the oral flora of individuals without halitosis [21]. Oral malodour has been treated with probiotics such as *Streptococcus salivarius*, *L. reuteri*, and *L. salivarius*; however, the specific process is unknown [16,13,23]. This in vitro research was designed to measure how well *L. rhamnosus* inhibits the growth of bacteria that cause bad breath.

## Materials and methods

The ethical consent was approved by the Mahatma Gandhi Mission’s Dental College and Hospital with reference number MGM/DCH/IEC/061/22. Strains of *P. intermedia* American Type Culture Collection (ATCC 25611), *P. gingivalis* (ATCC 33277), *T. forsythia* (ATCC 43037), and *L. rhamnosus* (ATCC 53103) were cultured and incubated in an anaerobic environment at 37°C. Study samples were divided into three groups: group A consists of the halitosis-causing bacterium *P. gingivalis* (ATCC 33277), group B consists of the halitosis-causing bacterium​​​​​​​ *T. forsythia* (ATCC 43037), and group C consists of the halitosis-causing bacterium *P. intermedia* (ATCC 25611). Each group has one species of bacterium.

Under anaerobic circumstances, 1 ml of a bacterial suspension (1 × 1089 cells/ml) in buffered saline of halitosis-causing pathogens was added to 10 ml of soft soy tryptic agar (0.7% agar). The first plate included 1 ml of a bacterial suspension (1 × 1089 cells/ml) of *P. gingivalis* added to 10 ml of soft soy tryptic agar (0.7% agar) (ATCC 33277). One millilitre of a bacterial solution containing *T. forsythia* (1 × 1089 cells/ml) was added to 10 ml of soft soy tryptic agar (0.7% agar) on plate 2 (ATCC 43037). One millilitre of a bacterial solution (1 × 1089 cells/ml) of *P. intermedia* was added to 10 ml of soft soy tryptic agar (0.7% agar) on plate 3 (ATCC 25611).

**Figure 1:**
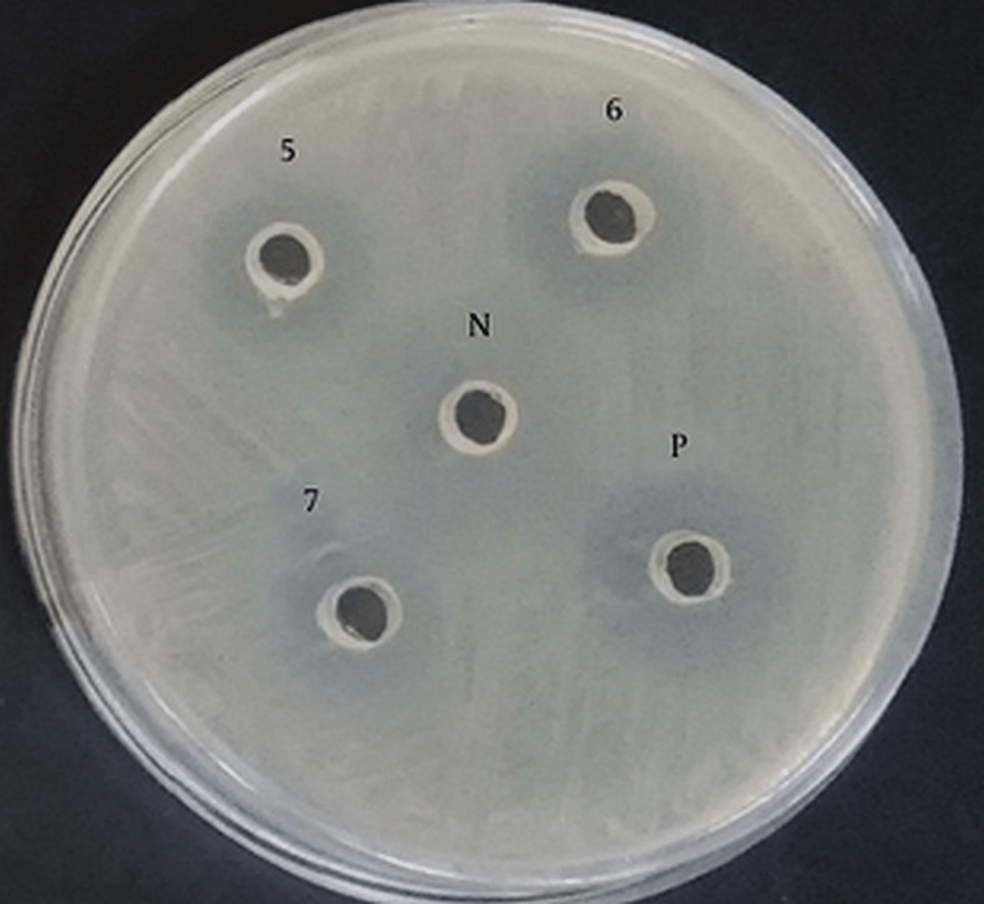
Lactobacillus rhamnosus well diffusion testing

Soy tryptic agar was inoculated at 30°C for three hours under ideal anaerobic conditions with three drops of a bacterial solution containing 1 × 1089 cells/ml of *L. rhamnosus* in buffered saline. Well diffusion tests were used to determine the extent to which *L. rhamnosus* (ATCC 53103) inhibited the growth of halitosis-causing bacteria for 24, 48, and 72 hours after droplet placement (Figure 1).

The information gathered was entered into an Excel spreadsheet and analysed using tools like SPSS (Statistical Programme for the Social Sciences). Descriptive statistics such as mean, standard deviation, median, and mode were used to summarise quantitative data, while frequency (n) and percentage (%) were used to summarise categorical data. The distribution of the numbers was analysed using the Kolmogorov-Smirnov and Shapiro-Wilk tests. The level of normality of the data was taken into account while deciding on the statistical tests to use. When comparing three or more groups using normally distributed continuous numeric data, a one-way analysis of variance (ANOVA) was used. The Kruskal-Wallis ANOVA was employed as a nonparametric alternative. Intragroup comparisons for numerical continuous data following a normal distribution were made using a paired t-test (for two observations) or repeated measures ANOVA for more than two observations; otherwise, a nonparametric substitute like the Wilcoxon signed rank test (for two observations) or Friedman’s test for more than two observations was used. Statistical significance was defined as p < 0.05 with an alpha error of 5%, a beta error of 20%, and 80% power.

## Results

The result of the present study showed that in group A (inhibitory effect of *L. rhamnosus* on *P. gingivalis*), there was a statistically significant variation in the frequencies across time periods (p = 0.01), with halo formation being more common after 72 hours and less common after 24 or 48 hours. The frequencies of halo formation at different time intervals in group B (inhibitory effect of *L. rhamnosus* on *T. forsythia*) were statistically significantly different from each other (p = 0.01), with a higher frequency for halo formation not seen at 24 hours and seen after 48 hours. There was a statistically significant difference between the frequencies for halo formation not seen at 24 hours but was observed after 48 hours in group C (inhibitory effect of *L. rhamnosus* on *P. intermedia*) (p = 0.01) (Table 1).

**Table 1:** Intergroup comparison * = statistically significant difference (p ≤ 0.05) ** = statistically highly significant difference (p ≤ 0.01) # = nonsignificant difference (p > 0.05)

Hours	Halo formation seen/not seen	Group A	Group B	Group C	Chi-square value	p value
At 24 hours	Not seen	11	11	11	-	-
At 48 hours	Not seen	11	4	3	13.933	0.001**
	Seen after 48 hours	0	7	8		
At 72 hours	Seen	0	0	8	13.933	0.001**
	Seen after 72 hours	11	11	3		

At 72 hours, there was a statistically significant difference in the frequencies among the groups (p = 0.01), with group A showing a higher frequency of halo formation than groups B and C. There was no longer a statistically significant difference in the frequency distributions between the groups after 48 hours (Table 2).

**Table 2:** Intragroup comparison * = statistically significant difference (p ≤ 0.05) ** = statistically highly significant difference (p < 0.01) # = nonsignificant difference (p > 0.05)

Groups	Halo formation seen/not seen	24 hours	48 hours	72 hours	Chi-square value	p value
Group A	Not seen	11	11	0	33.000	0.001**
	Seen after 72 hours	0	0	11		
Group B	Not seen	11	4	0	48.400	0.001**
	Seen	0	0	7		
	Seen after 48 hours	0	7	0		
	Seen after 72 hours	0	0	4		
Group C	Not seen	11	3	0	51.857	0.001**
	Seen	0	0	8		
	Seen after 48 hours	0	8	0		
	Seen after 72 hours	0	0	3		

## Discussion

In recent years, the notion of biological plaque management has emerged, which involves the use of probiotics and vaccinations to eradicate disease-causing bacteria. Yet, choosing the optimal probiotic for dental health is a contentious matter. It was decided that the probiotic would be *L. rhamnosus* SP1 [24] since this strain has been shown to have positive effects on the immune responses of both children and adults. In healthy individuals without baseline periodontopathogens, the use of *L. rhamnosus* GG tablets reduced gingival aggravation [15]. This was seen without any change to the oral microbiome.

This in vitro research compares the effectiveness of *L. rhamnosus* against germs that cause bad breath. The results indicated that after 72 hours, *L. rhamnosus* had an inhibitory impact on *P. gingivalis* in all 11 samples from group A and on *T. forsythia* in five samples from group B. After 48 hours, inhibitory effects were shown in seven of the 11 samples tested for group A and eight of the 11 samples tested for group C, where *P. intermedia* was inhibited by *L. rhamnosus*.

*P. gingivalis*, *P. intermedia*, *F. nucleatum*, and *T. forsythia* all produce VSCs, which contribute to bad breath [19,20]. The purpose of this research was to determine whether the probiotic *L. rhamnosus* inhibited the development of halitosis-causing bacteria such as *P. gingivalis*, *P. intermedia*, and *T. forsythia*. The consequence of the review is like that of Mousquer et al., who found that the probiotic *L. salivarius* G60 restrained the development of *P. gingivalis* and *P. intermedia*, supporting the prevalence of *Lactobacillus* + inulin in diminishing halitosis by the creation of lactic corrosive, hydrogen peroxide, and bacteriocins or bacteriocin-like substances that might act alone or in showing hindering microorganisms [24].

The effect of probiotics on bacterial infections is due to the production of antibacterial substances like bacteriocin. Bacteriocins are a diverse group of short, heat-stable peptides that exhibit strong antibacterial action by disrupting the structure and production of cell walls, forming pores in the bacterial membrane. Although the production of bacteriocins by *L. rhamnosus* is still not clear, *L. rhamnosus* has been shown to have bactericidal properties. Among the seven peptides, the isolated NPSRQERR peptide was considered to have the most potent antibacterial effect [25].

According to Kazor et al., *S. salivarius* species can produce bacteriocins that help reduce the prevalence of bacteria like *Atopobium parvulum*, *Eubacterium sulci*, and *Fusobacterium*, which are commonly linked to halitosis and produce VSCs responsible for the odour [26]. The use of *S. salivarius* K12 gum or lozenges resulted in a decrease in VSCs, as reported by Burton et al. in 2006 [27]. Researcher Kang et al. found that gargling with a *Weissella cibaria*-containing probiotic solution significantly reduced the amount of VSCs generated by *F. nucleatum* [28]. Within the limitations of the study, the inhibitory effect was seen only for a period of 72 hours; more studies with a longer duration are required. Also, there is no evidence to support the claim that *L. rhamnosus* produces bacteriocins that must have caused the inhibitory effect on halitosis-causing bacteria.

## Conclusions

In the present study, the zones of inhibitory halo formation of *L. rhamnosus* on *P. gingivalis* were seen after 72 hours, which showed a statistically significant difference; similarly, for *T. forsythia* and *P. intermedia*, a statistically significant difference was seen after 48 hours. The authors conclude that the bacteria responsible for bad breath, such as *P. gingivalis*, *T. forsythia*, and *P. intermedia*, are inhibited by *L. rhamnosus*. Further research with a larger sample size and over a longer period of time is required to screen for additional types of halitosis-causing bacteria.
